# Comparison of PK/PD Targets and Cutoff Values for Danofloxacin Against *Pasteurella multocida* and *Haemophilus parasuis* in Piglets

**DOI:** 10.3389/fvets.2022.811967

**Published:** 2022-02-02

**Authors:** Yu-Feng Zhou, Zhen Sun, Rui-Ling Wang, Jian-Guo Li, Chao-Yan Niu, Xian-An Li, Yun-Yun Feng, Jian Sun, Ya-Hong Liu, Xiao-Ping Liao

**Affiliations:** ^1^National Risk Assessment Laboratory for Antimicrobial Resistance of Animal Original Bacteria, College of Veterinary Medicine, South China Agricultural University, Guangzhou, China; ^2^Guangdong Laboratory for Lingnan Modern Agriculture, Guangzhou, China; ^3^Guangdong Provincial Key Laboratory of Veterinary Pharmaceutics Development and Safety Evaluation, South China Agricultural University, Guangzhou, China

**Keywords:** PK/PD, cutoff, danofloxacin, *P. multocida*, *H. parasuis*

## Abstract

Danofloxacin is a synthetic fluoroquinolone with broad-spectrum activity developed for use in veterinary medicine. The aim of this study was to evaluate the pharmacokinetic/pharmacodynamic (PK/PD) targets, PK/PD cutoff values and the optimum doses of danofloxacin against *P. multocida* and *H. parasuis* in piglets. Single dose serum pharmacokinetics was determined in piglets after intravenous and intramuscular administration of 2.5 mg/kg. Danofloxacin was well absorbed and fully bioavailable (95.2%) after intramuscular administration of 2.5 mg/kg. The epidemiological cutoff (ECOFF) values of danofloxacin from 931 *P. multocida* isolates and 263 *H. parasuis* isolates were 0.03 and 4 mg/L, respectively. Danofloxacin MICs determined in porcine serum were markedly lower than those measured in artificial broth, with a broth/serum ratio of 4.33 for *H. parasuis*. Compared to *P. multocida*, danofloxacin exhibited significantly longer post-antibiotic effects (3.18–6.60 h) and post-antibiotic sub-MIC effects (7.02–9.94 h) against *H. parasuis*. The mean area under the concentration-time curve/MIC (AUC_24h_/MIC) targets of danofloxacin in serum associated with the static and bactericidal effects were 32 and 49.8, respectively, for *P. multocida*, whereas they were 14.6 and 37.8, respectively, for *H. parasuis*. Danofloxacin AUC_24h_/MIC targets for the same endpoints for *P. multocida* were higher than those for *H. parasuis*. At the current dose of 2.5 mg/kg, the PK/PD cutoff (CO_PD_) values of danofloxacin against *P. multocida* and *H. parasuis* were calculated to be 0.125 and 0.5 mg/L, respectively, based on Monte Carlo simulations. The predicted optimum doses of danofloxacin for a probability of target attainment (PTA) of > 90% to cover the overall MIC population distributions of *P. multocida* and *H. parasuis* in this study were 2.38 and 13.36 mg/kg, respectively. These PK/PD-based results have potential relevance for the clinical dose optimization and evaluation of susceptibility breakpoints for danofloxacin in the treatment of swine respiratory tract infections involving these pathogens.

## Introduction

*P. multocida* and *H. parasuis* play important roles in many outbreaks of swine respiratory disease (SRD) and act together to increase the severity and duration of lung damage caused by other symbiotic viruses and bacteria such as porcine circovirus type 2 and *Streptococcus suis* (1–3). Furthermore, studies indicated that *P. multocida* type A can act as the primary pathogen of porcine pneumonia and septicemia with a rising prevalence rate reported from 8% to 15.6% in China, Korea and United States (1, 4, 5). Due to the high prevalence of mixed infections with multiple bacterial species, the treatment of SRD generally includes the use of broad-spectrum antibiotics (6, 7). Fluoroquinolones, such as danofloxacin, possess excellent PK characteristics that may contribute to clinical success of treating SRD. Such advantages include high peak concentrations in plasma, extensive distribution to most tissues in animal body and deep penetration into lung fluids (8, 9). Despite these findings from previous studies, the precise pharmacokinetic/pharmacodynamic (PK/PD) targets and cutoff values of danofloxacin in pigs for SRD pathogens, especially for *P. multocida* have not been fully elucidated.

This study sought to determine and compare the PK/PD relationships of danofloxacin between *P. multocida* and *H. parasuis* with the goal to provide a framework for further study and optimization of danofloxacin dosing strategies for the treatment of bacterial respiratory mixed infections in piglets caused by SRD pathogens. By evaluating the drug kinetics, PK/PD targets, post-antibiotic effect (PAE) and postantibiotic sub-MIC effect (PA-SME), the optimum doses and PK/PD cutoffs (CO_PD_) of danofloxacin were estimated for *P. multocida* and *H. parasuis*. A comparison of these results provides insights into the accurate antibiotic treatment for SRD.

## Materials and Methods

### Bacterial Strains and Susceptibility Testing

The MIC distribution of 931 swine-origin *P. multocida* isolates (Supplementary Table 1) was obtained by merging data from our own laboratory and some previous studies (10, 11). A total of 263 isolates of *H. parasuis* were gathered during 5-year surveillance study in different provinces of China from 2015 to 2020 (Supplementary Table 1). All the isolates were collected from diseased pigs suffering polyserositis, pneumonia or arthritis, and cultured with Haemophilus test medium (HTM) broth and agar containing 20 mg/L β-NAD and 5% lysed horse blood. Bacterial species was identified using the Axima Assurance MALDI-TOF mass spectrometer (Shimadzu Corp., Kyoto, Japan), as previously described (12). The MICs of danofloxacin for these isolates were determined using the broth microdilution in accordance with CLSI guidelines (13). To determine if there is a potentiation effect of serum on susceptibility, the danofloxacin MICs against *P. multocida* and *H. parasuis* were further determined in both broth and porcine serum.

### Epidemiological Cutoff Values Determination

The ECOFF defines the upper end of the wild-type MIC distribution devoid of phenotypically detectable acquired resistance mechanisms (14). The isolates carrying plasmid-mediated quinolone resistance (PMQR) genes [*qnrA-D, qepA, oqxAB* and *aac(6')-Ib-cr*] were consequently removed (15). The log_2_-transformed MIC distribution of danofloxacin for *P. multocida* and *H. parasuis* was subjected to the statistical goodness-of-fit and non-linear least-squares regression tests to obtain optimum normal distribution (16). The final ECOFF value was calculated as the MIC that captured at least 95% of the optimum MIC distribution using the ECOFFinder program (16, 17).

### PAEs and PA-SMEs Determination

Two *P. multocida* and three *H. parasuis* strains were selected to expose to danofloxacin at 1 × and 4 × MICs for 1 h. After removal of drug by centrifugation at 3,000 g for 10 min, bacterial cells were resuspended in drug-free broth (PAE) and broth containing 0.1 to 0.3 × MICs of danofloxacin (PA-SME) for continuous measurement of the absorbance at 600 nm. Optical density was converted into bacterial counts using a standard curve, as our previously reported (17). The PAEs and PA-SMEs of danofloxacin against *P. multocida* and *H. parasuis* were calculated as follow: PAE/PA-SME = T/T_PA_-C, where C is the time for 1-log_10_ control growth and T/T_PA_ is the time for 1-log_10_ growth after drug removal (T) or in the sub-MIC treated phase (T_PA_) (18).

### Pharmacokinetics of Danofloxacin in Piglets

Twelve healthy crossbred piglets (Duroc × Landrace × Yorkshire, 9.3 ± 1.9 kg from Jiahe Agricultural Stockbreeding Co., Qingyuan, China) were used for a two-period crossover study. Animal experimental protocols were approved by the Animal Ethics Committee of South China Agricultural University (approval no. 2018014). Each piglet received danofloxacin (Injectable solution; lot no. 190201; Hainan Yuqi Pharmaceutical Co., Dingan, China) at a dose of 2.5 mg/kg b.w. by intravenous (IV) and intramuscular (IM) injections. Feed and water were provided *ad libitum*. The dose of danofloxacin was chosen based on previous PK studies in pigs and the manufacturer's instruction (19–22). The serums samples for danofloxacin concentration determination were collected from the jugular veins into vacutainers without anticoagulant prior to dosing (0 h) and at 0.08, 0.25, 0.5, 0.75, 1, 2, 4, 6, 8, 12, 24, 36, and 48 h after administrations of danofloxacin.

Danofloxacin concentrations in serum samples were measured by a liquid chromatography-tandem mass spectrometry (LC-MS/MS) method (details are given in Supplementary Data Sheet 1). All PK parameters were calculated using the compartmental models in WinNonlin software (version 5.2; Pharsight, St. Louis, MO, USA). The Akaike information criterion (AIC) was used to guide the selection of the best PK model to describe the observed time-concentration data. Danofloxacin average bioavailability (F%) after intramuscular injection was calculated by dividing each AUC_IM_ value by their respective AUC_IV_ value for each individual piglet according to the following standard equation (23): F% = AUC_infinity(IM)_ / AUC_infinity(IV)_ ×100%.

### *Ex vivo* Time-Kill and PK/PD Index Target for Efficacy

The abilities of danofloxacin to kill *P. multocida* and *H. parasuis* were assessed *ex vivo* as previously described (17). Serum samples collected from piglets at different time points were filtered to avoid bacterial contamination. Bacterial cells were subcultured and inoculated to each serum sample, giving an initial inoculum of ~10^6^cfu/mL. The mixtures were serially diluted and plated using a drop-plate technique to enumerate bacterial CFUs after 3, 6, 9 and 24 h of incubation. The limit of detection (LOD) was 40 cfu/mL.

The correlation between antibacterial efficacy and the PK/PD parameter AUC_24h_/MIC was determined by the non-linear WinNonlin regression program (version 5.2; Pharsight, St. Louis, MO, USA). The AUC/MIC ratio was chosen as the predictive PK/PD parameter as previous studies have demonstrated this index to be predictive for fluoroquinolones (24, 25). The sigmoid *E*_max_ model used was derived from the Hill equation: *E* = *E*_0_ + *E*_max_ × *C*^*N*^ / (*EC*_50_^*N*^ + *C*^*N*^), where *E*_0_ is the log_10_ change of bacterial count in the absence of danofloxacin, *E*_max_ is the maximum effect, *C* is the PK/PD index (AUC_24h_/MIC), *EC*_50_ is the AUC_24h_/MIC required to achieve 50% of the *E*_max_ and *N* is the slope of the dose-response curve. The coefficient of determination (R^2^) was used to estimate the variance due to regression with the PK/PD parameter AUC/MIC. The AUC_24h_/MIC targets in serum required to produce bacteriostatic (E = 0), bactericidal (E = −3) and eradication (E = −4) effects were calculated for each drug-organism combination.

### PK/PD Cutoff Determination and Dose Assessment

Based on PK parameters and calculated PK/PD targets (AUC_24h_/MIC) for bactericidal effect, a 10,000-subject Monte Carlo simulation was conducted to obtain the danofloxacin PK/PD cutoffs (CO_PD_) for *P. multocida* and *H. parasuis* using Crystal Ball software (version 11.1.2, Oracle Corporation) (26). The AUC_24h_/MIC was calculated with the following formula: AUC_24h_/MIC = Dose / (Cl × MIC). Clearance (Cl) was assumed to be normally distributed in the form of mean ± SD (Table 1). Scenarios were simulated separately at each possible MIC. The CO_PD_ was defined as the highest MIC at which the PTA was ≥90% (27).

**Table 1:** The PK parameters of danofloxacin in porcine serum following single dose intravenous (IV) and intramuscular (IM) administrations at 2.5 mg/kg.

**PK parameters*^a^***	**Unit**	**IV route**	**IM route**
K_a_	1/h		4.99 ± 5.08
K_el_	1/h		0.21 ± 0.12
A	mg/L	1.12 ± 0.68	–
α	1/h	4.44 ± 2.24	–
B	mg/L	1.67 ± 0.89	–
β	1/h	0.20 ± 0.05	–
T_1/2Kel_	h		4.18 ± 1.81
T_1/2Ka_	h		0.29 ± 0.20
T_1/2α_	h	0.25 ± 0.24	–
T_1/2β_	h	3.76 ± 1.00	–
V_ss_	L/kg	1.90 ± 1.22	–
Cl	L/kg/h	0.39 ± 0.27	–
AUC_infinity_	mg·h/L	9.39 ± 5.79	8.17 ± 3.51
T_max_	h		1.04 ± 0.52
C_max_	mg/L		1.19 ± 0.55
F	%	–	95.2 ± 21.9

a *K_a_, constant of absorption rate; K_el_, constant of elimination rate; A, intercept for the distribution phase; α, distribution rate constant; B, intercept for the elimination phase; β, elimination rate constant; T_1/2Kel_, elimination half-life in the one-compartment model; T_1/2Ka_, absorption half-life; T_1/2α_, distribution half-life; T_1/2β_, elimination half-life in the two-compartment model; V_ss_, volume of distribution at steady state; Cl, systemic clearance; AUC_infinity_, the area under the concentration-time curve from zero to infinity; T_max_, time to reach the peak concentration (C_max_); F, average bioavailability*.

In order to ascertain the optimum dose regimens of danofloxacin to cover the overall MIC population distributions in this study (931 *P. multocida* strains and 263 *H. parasuis* isolates), the two population distributions of danofloxacin doses were predicted by a 10,000-subject Monte Carlo simulation. The dose was calculated by the equation as follow (17, 28): Dose = (Cl × AUC/MIC × MIC_distribution_) / (*fu* × F), where Cl is the body clearance; AUC/MIC is the PK/PD target required for a bactericidal effect, in this case, the AUC_24h_/MIC of 49.8 and 37.8 for *P. multocida* and *H. parasuis*, respectively; a scaling factor of 4.33 was used to bridge the MIC differences between HTM and serum when dose distribution was predicted for *H. parasuis*; *fu* is free drug fraction using protein binding rate of 44% in porcine serum (29); F is the bioavailability of IM administration.

## Results

### MICs and ECOFF Determination

**Figure 1:**
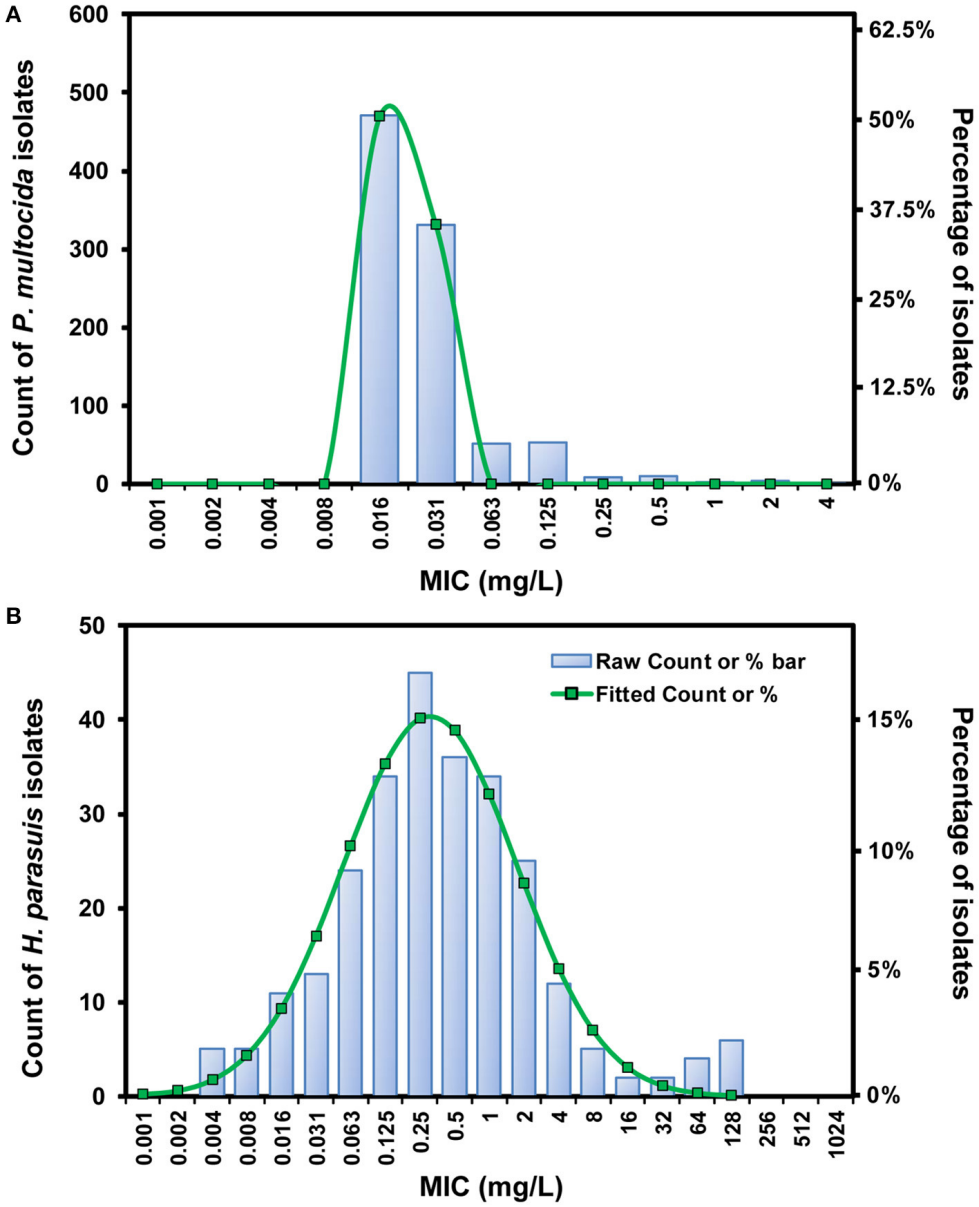
The log_2_-transformed MIC distribution of danofloxacin against swine-origin *P. multocida* **(A)** and *H. parasuis* **(B)**. The MIC distribution of *P. multocida* isolates (*n* = 931) was created by merging data from our laboratory and some previous studies (10, 11). *H. parasuis* isolates (*n* = 263) and MIC data were obtained in our own laboratory from 2015 to 2020. The number of isolates and the observed frequency corresponding to each MIC value are shown along the y-axes. The lines represent predicted frequency based on the best fitting log_2_-normal distribution [log_2_ mean (−6.06) ± SD (0.27) for *P. multocida* and log_2_ mean (−2.21) ± SD (2.49) for *H. parasuis*, respectively].

Of 931 *P. multocida* isolates, the fitted MIC distribution [Log_2_ mean (−6.06) ± SD (0.27)] contained > 95% that possessed danofloxacin MICs ≤ 0.03 mg/L, and the ECOFF was consequently calculated to be 0.03 mg/L for *P. multocida* (Figure 1A). No difference in MIC was observed for *P. multocida* between broth and serum. However, of the 14 *H. parasuis* isolates tested, geometric mean of danofloxacin MIC in serum was significantly lower than that in HTM, with a HTM/serum ratio of 4.33 (*P* < 0.05; Supplementary Figure 1). The MICs of danofloxacin against our 263 clinical *H. parasuis* isolates ranged from 0.004 to 128 mg/L in HTM, with the MIC_50_ and MIC_90_ of 0.25 and 4 mg/L, respectively (Supplementary Table 1). In order to obtain a unimodal distribution, the 10 isolates with MICs of ≥64 mg/L were therefore removed. More than 95% of the best fitting normal distribution [Log_2_ mean (−2.21) ± SD (2.49)] was in the range of 0.004 to 4 mg/L, thus the ECOFF value was determined to be 4 mg/L for *H. parasuis* (Figure 1B).

### PAEs and PA-SMEs

**Figure 2:**
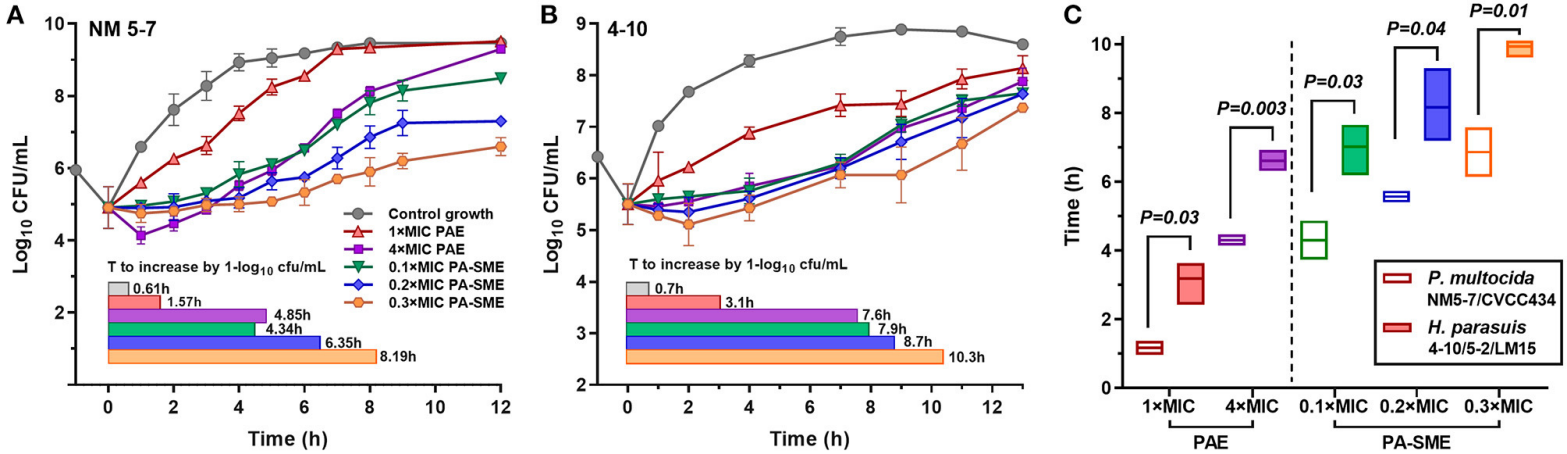
The postantibiotic effects (PAEs) and post-antibiotic sub-MIC effects (PA-SMEs) of danofloxacin. **(A,B)** The PAEs were measured after initial exposure to danofloxacin at 1 × and 4 × MICs against *P. multocida* (A; strain NM5-7) and *H. parasuis* (B; strain 4–10). The PA-SMEs were measured after initial exposure to danofloxacin at 4 × MICs. The color horizontal bars represent the time that required bacterial counts to increase by 1.0-log_10_cfu/mL after drug removal (PAE) or at the sub-MIC phase (PA-SME). **(C)** Comparison of danofloxacin PAE and PA-SME between *P. multocida* and *H. parasuis*. Two *P. multocida* isolates and three *H. parasuis* isolates were tested and included. Statistical significance was determined using the two-tailed unpaired Student *t-test* (*P* < 0.05).

PAEs were calculated after removal of bacterial cells from danofloxacin exposures at 1 × and 4 × MICs. Persistent regrowth inhibition was observed in a concentration-dependent manner, resulting in PAE values of 0.96–4.46 h for *P. multocida* and 2.42–6.92 h for *H. parasuis*, respectively (Figures 2A,B). The addition of sub-MIC danofloxacin during the post-antibiotic phase substantially delayed bacterial regrowth, producing PA-SMEs of 4.30–6.86 h for *P. multocida* and 7.02–9.94 h for *H. parasuis*, respectively (Figure 2C). Despite this fact, the mean bacterial densities in the presence of sub-MIC danofloxacin remained at a lower level than their respective growth controls until at least 12 h (Figure 2; Supplementary Figure 2). Of note, the time suppression of regrowth (PAE and PA-SME) for *H. parasuis* was significantly longer relative to *P. multocida* (*P* < 0.05; two-tailed unpaired Students *t-test*; Figure 2C).

### Danofloxacin PKs in Piglets

**Figure 3:**
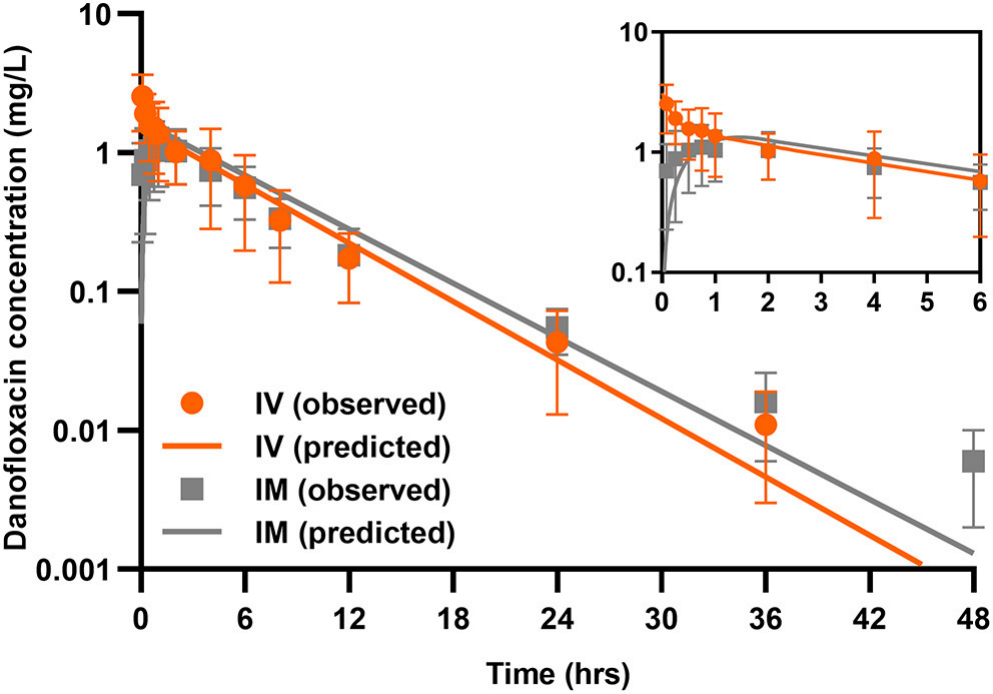
Danofloxacin pharmacokinetics in piglets. Serum concentrations of danofloxacin following single dose intravenous (IV) and intramuscular (IM) administrations at 2.5 mg/kg in piglets (*n* = 12 per time point). Upper right panel shows details from 0 to 6 h. The lines drawn through the time points indicated the best-fitting lines based on the compartment models.

A two-compartmental model fit was shown for time-concentration profile of danofloxacin after IV injection (Figure 3), which was consistent with previous results observed in both healthy and infected pigs (29). Notably, the decline in serum danofloxacin concentrations was bi-exponential with half-lives of (T_1/2α_) 0.25 h and (T_1/2β_) 3.76 h for distribution and elimination phases, respectively (Table 1). After IM dosing of danofloxacin, the mean peak concentration (C_max_; 1.19 mg/L) was reached in serum within 1.04 h. While the prolonged terminal half-life (T_1/2Kel_; 4.18 h) was observed in serum following IM dosing, AUC_infinity_ values were comparable regardless of administration routes, indicating a high bioavailability of 95.2% after IM administration (Table 1).

### *Ex vivo* Antimicrobial Activities and PK/PD Targets

**Figure 4:**
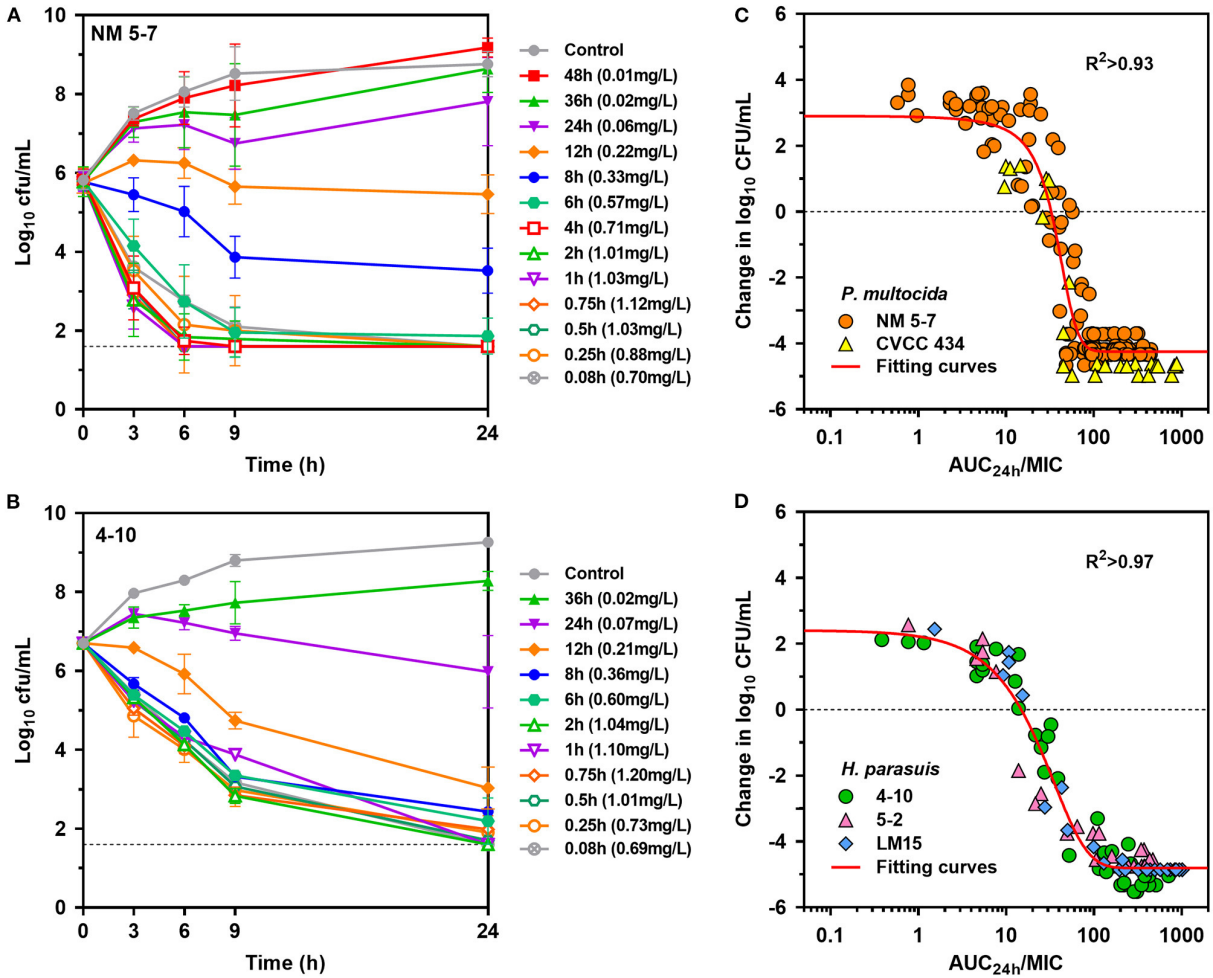
*Ex vivo* activity and PK/PD relationships of danofloxacin. **(A,B)** *Ex vivo* time-kill curves of danofloxacin against *P. multocida* [**(A)** strain NM5-7; MIC_serum_ = 0.13 mg/L] and *H. parasuis* [**(B)** strain 4-10; MIC_serum_ = 0.06 mg/L] in serums of piglets receiving intramuscular injection of danofloxacin (2.5 mg/kg b.w.). Numerical values on right brackets represent the mean concentrations of danofloxacin in serums collected from different time points post-dosing. **(C,D)** Correlation plots between *ex vivo* activity and AUC_24h_/MIC of danofloxacin using the sigmoid E_max_ equation. The fitting curves represent predicted values (two *P. multocida* and three *H. parasuis* included), and the points represent values of individual serum samples collected from 0 to 48 h.

Rapid activity against *P. multocida* strain NM5-7 (MIC_serum_ = 0.13 mg/L) was demonstrated with porcine serum collected up to 12 h at concentrations of 0.22 to 1.12 mg/L (Figure 4A). Notably, a concentration-dependent trend toward a greater level of *P. multocida* killing was observed with increasing danofloxacin concentrations in serums. Bacterial densities of *P. multocida* were driven below detectable limits by serums collected up to 6 h after 9 h of incubation (Figure 4A). Concentration-dependent killing activity was similarly observed for all *H. parasuis* strains tested (Figure 4B; Supplementary Figure 3). Accordingly, *ex vivo* activity was negligible for serums at 36 and 48 h, while sustained bactericidal activity was attained within 9 h of exposure to serums containing danofloxacin > 0.5 mg/L (Supplementary Figure 3). For *H. parasuis* 4–10 (MIC_serum_ = 0.06 mg/L), complete bactericidal activities reaching undetectable limits of eradication were noted within 24 h in response to serums collected up to 12 h (Figure 4B).

PK/PD analyses of the *ex vivo* time-kill data were performed to determine AUC/MIC targets of danofloxacin associated with the optimal activity. PK/PD relationships between AUC/MIC and *ex vivo* activity were strong, with an R^2^ of > 0.93 (Figures 4C,D). For *P. multocida*, the mean AUC/MIC targets in serum for bacteriostatic, bactericidal and eradication effects were 32, 49.8 and 66.9, respectively. Of note, the PK/PD targets for *H. parasuis* were much lower than those for *P. multocida* (*P* < 0.05, two-tailed unpaired Student's *t*-test). Serum AUC/MIC targets for the same endpoints were 14.6, 37.8 and 62.9 (Table 2).

**Table 2:** PK/PD targets of danofloxacin in serum (AUC_24h_/MIC) necessary to achieve the bacteriostasis, bactericidal, and eradication effects for the study organisms in piglets.

**Organisms**	**MIC in serum (mg/L)**	***E*_0_**	***E*_max_**	***EC*_50_**	***N***	**R^2^**	**Target values of AUC_24h_/MIC ratio (h) in serum *^a^***		
							**Bacteriostasis**	**Bactericidal**	**Eradication**
*P. multocida*									
NM5-7	0.125	2.95	−4.29	38.9	3.13	0.93	33.1	58.7	86.8
CVCC434	0.031	1.29	−4.69	36.7	8.20	0.96	30.9	40.9	47.1
**Mean**	**NA**	**2.12**	–**4.49**	**37.8**	**5.67**	**NA**	**32.0**	**49.8**	**66.9**
**SD**	**NA**	**0.83**	**0.20**	**1.10**	**2.54**	**NA**	**1.10**	**8.91**	**19.8**
*H. parasuis*									
4–10	0.063	2.05	−5.24	31.8	1.64	0.97	16.8	50.5	79.3
5–2	0.063	2.88	−4.59	12.8	1.80	0.98	10.2	24.6	50.5
LM15	0.031	1.91	−4.83	25.4	2.31	0.97	16.8	38.4	58.9
**Mean**	**NA**	**2.28**	–**4.89**	**23.3**	**1.92**	**NA**	**14.6**	**37.8**	**62.9**
**SD**	**NA**	**0.43**	**0.27**	**7.89**	**0.29**	**NA**	**3.11**	**10.5**	**12.1**

a *The bacteriostasis, bactericidal and eradication effects were defined as the net static, 3-log_10_, and 4-log_10_ kill endpoints over 24 h; P < 0.05 for bacteriostasis AUC/MIC target between P. multocida and H. parasuis (unpaired Student's t-test); Bold values indicate the means and standard deviations (SD); NA, not applicable*.

### PK/PD Cutoff Determination and Dose Prediction

**Figure 5:**
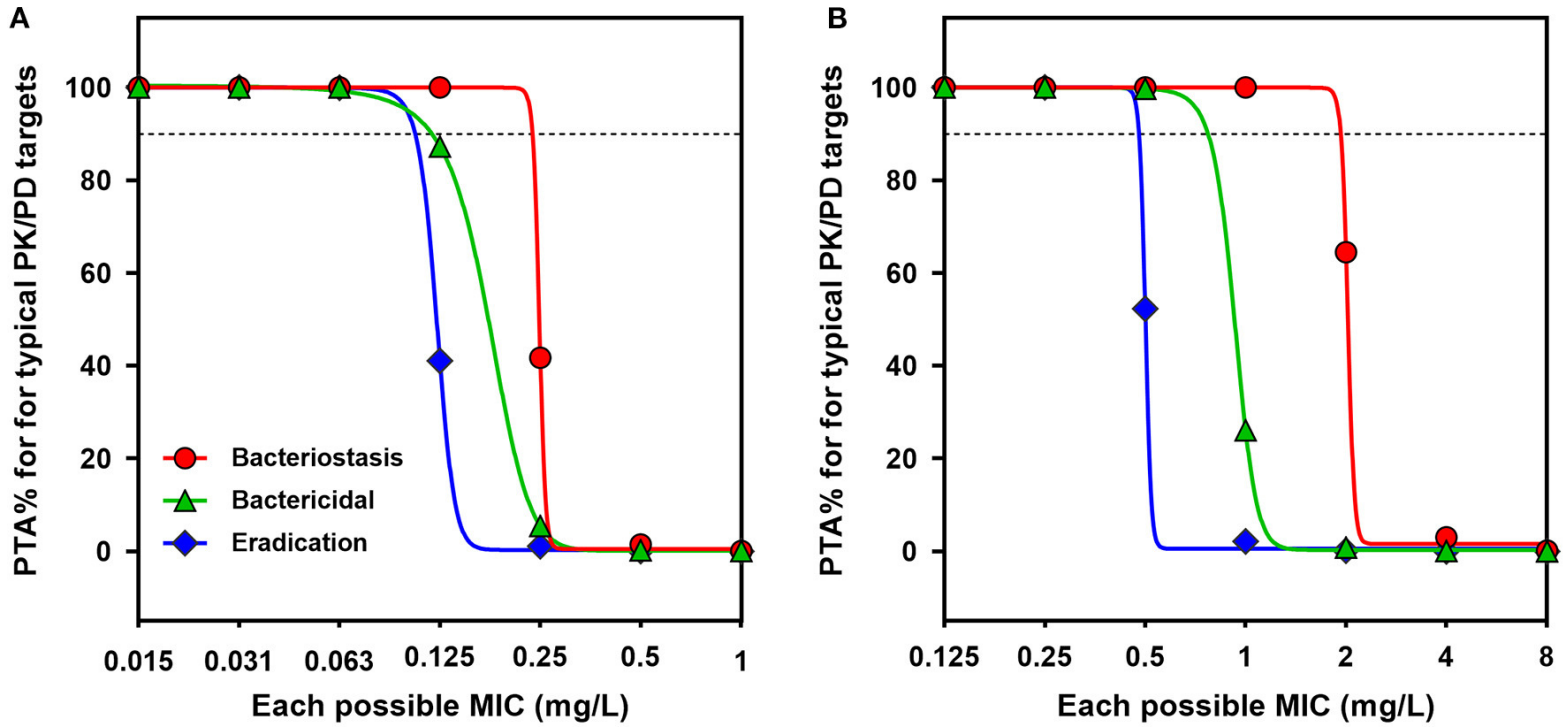
PK/PD cutoff values for danofloxacin against *P. multocida* and *H. parasuis*. **(A,B)** Probability of target attainment (PTA) for typical AUC_24h_/MIC targets (bacteriostatic, bactericidal and eradication effects) at each possible MIC when treated with danofloxacin at dose of 2.5 mg/kg against *P. multocida* **(A)** and *H. parasuis* **(B)** infections. Dotted lines denote the PTA of 90%.

The probabilities of the current dose regimen (2.5 mg/kg) achieving typical AUC/MIC targets at each possible MIC were determined by a 10,000-iteration Monte Carlo simulation, from which PTAs were estimated (Figure 5). With a target AUC/MIC ratio of 49.8 (i.e., bactericidal action for *P. multocida*), the PTA was still 87.3% at a MIC of 0.125 mg/L. The CO_PD_ value of danofloxacin for *P. multocida* was consequently determined to be 0.125 mg/L (Figure 5A). In view of the significant potentiation effect of serum on activity of danofloxacin for *H. parasuis* (Supplementary Figure 1), a scaling factor of 4.33 was created to bridge the MIC variation between HTM and serum when calculating the CO_PD_ for *H. parasuis*. The PTA for AUC/MIC ratio of 37.8 was only 26.1% at a MIC of 1.0 mg/L and reached 99.7% when the MIC was 0.5 mg/L. The CO_PD_ of danofloxacin against *H. parasuis* was therefore defined as a MIC of 0.5 mg/L (Figure 5B).

**Figure 6:**
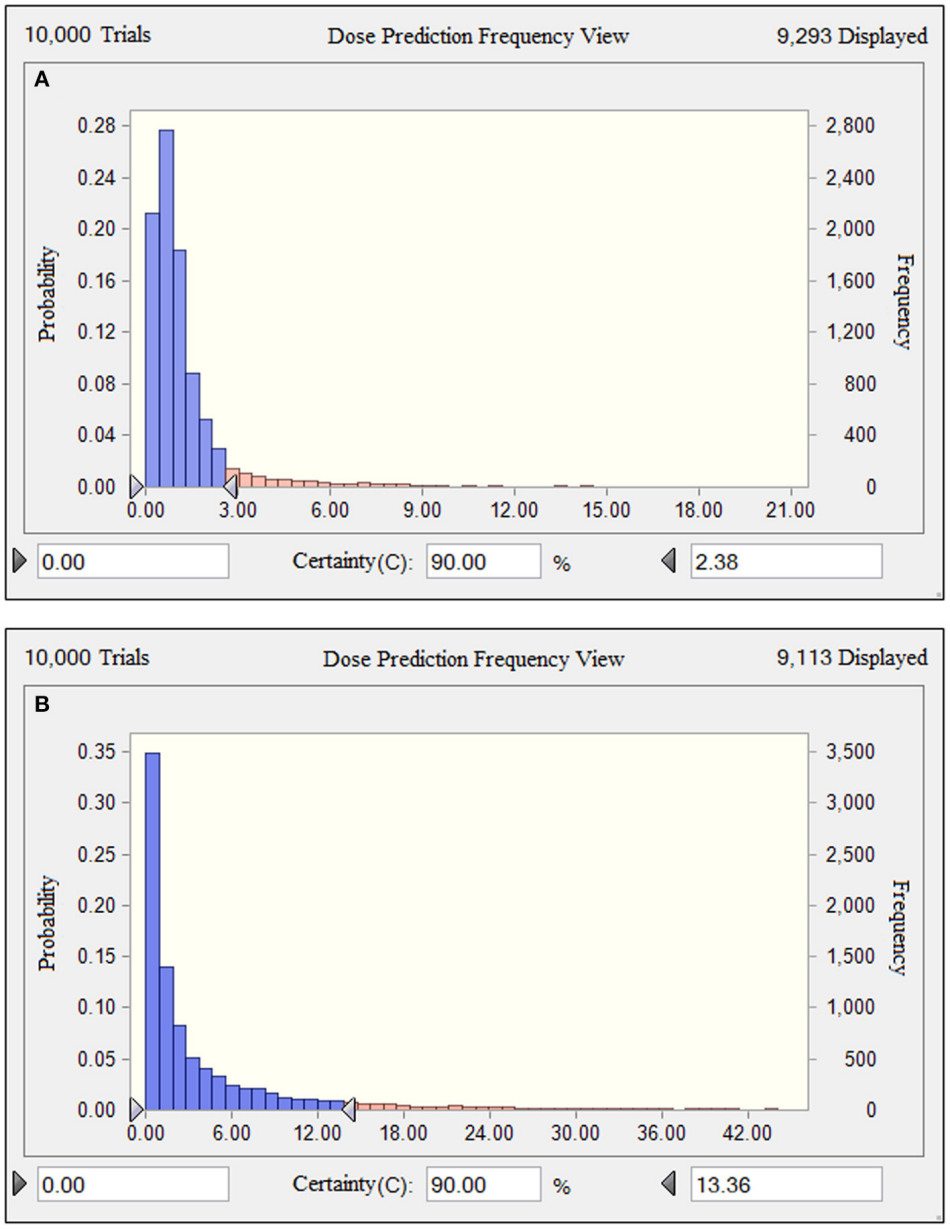
Comparison of population distributions of danofloxacin doses for *P. multocida* **(A)** and *H. parasuis* **(B)**. The right triangle depicted the calculated target doses for a PTA of > 90% to cover the overall MIC population distributions in this study.

Based on the results of the current PK parameters, PK/PD targets and the MIC distribution, if danofloxacin was given once daily intramuscularly in piglets, the predicted dosages for a PTA of 90% to cover the overall MIC population distributions in this study were 2.38 and 13.36 mg/kg (Figure 6), which were estimated to be effective achieving a bactericidal effect against *P. multocida* and *H. parasuis*, respectively.

## Discussion

In this study, we observed a marked potentiation effect of porcine serum on danofloxacin activities for *H. parasuis* but not for *P. multocida*. This finding was in agreement with the previous studies showing that incorporation of the increasing amounts of serum to broth progressively reduced macrolides MICs for seven bacterial species harvested from pigs (28, 30). On the contrary, reduced antimicrobial activity in serum was observed for moxifloxacin against *Staphylococcus aureus* (31). It is therefore likely that serum potentiation effect is strain- and drug-codependent (32). The complement and specific antibody are considered as the key factors responsible for increased serum activity (33). In general, a prolong PAE has a high likelihood of predicting favorable outcomes (34). The findings of our study exhibiting the notable PAEs from 0.96 to 6.92 h in a concentration-dependent manner for both *P. multocida* and *H. parasuis*, point to clinical treatment with fluoroquinolones that cloud be administered at a longer dosing interval without loss of efficacy. It is well known that fluoroquinolones disrupt DNA synthesis by binding bacterial gyrase and topoisomerase, the PAEs induced by danofloxacin may represent the lag time for drug to dissociate from binding sites and to diffuse out of bacteria (35).

Similar to other fluoroquinolones, danofloxacin have good penetration into pulmonary epithelial lining fluid (ELF). A previous bronchopulmonary PK study with danofloxacin demonstrated higher peak concentration in ELF compared to plasma in pigs, with a mean ELF/plasma AUC ratio of 5.4 (9). Of note, the pharmacokinetic profiles of danofloxacin were linear and proportional in piglets as described by the result of linear regression analysis (R^2^ = 0.951 for AUC_24h_) (36). The similarity and proportionality of PK profiles potentially reflects passive diffusion of danofloxacin from plasma to ELF. In this case, serum could be used as a predictive surrogate for PD target assessment, although the value of PD target could be relatively high (37). The PD targets associated with bactericidal action in previous fluoroquinolone studies has been a total AUC_24h_/MIC of 88 for *H. parasuis*, and values 1.5- to 5-fold higher (121–451) for gram-negative pathogens such as *Escherichia coli* and *Salmonella typhimurium* (38–40). In our study with danofloxacin, the PD targets were lower for each of the bacteria tested. This difference was most profound for *H. parasuis* with a mean bactericidal AUC_24h_/MIC of 37.8. Similarly, the AUC_24h_/MIC target identified for *P. multocida* (49.8) was modestly lower than marbofloxacin with a bactericidal AUC_24h_/MIC of 64.9 (41). These endpoints were roughly 2- to 9-fold lower than comparative PD studies for veterinary fluoroquinolones. The notable PK/PD efficacies for both *H. parasuis* and *P. multocida* provide a fairly robust option for treating SRD, especially in situations of bacterial coinfections due to mixed species.

At the current clinical dose of 2.5 mg/kg, the ECOFF and CO_PD_ values of danofloxacin against *P. multocida* were determined to be 0.03 and 0.125 mg/L, respectively. This is similar with the EUCAST MIC breakpoint (0.06 mg/L) used for levofloxacin and ciprofloxacin against *P. multocida* (42). The clinical trial has been previously conducted to investigate danofloxacin efficacy in Danish swine herds with a naturally occurring outbreak of acute *Pasteurella* pneumonia. A satisfactory response to treatment with 1.25 mg/kg danofloxacin was observed in 87% of the diseased pigs (43). For *H. parasuis*, danofloxacin CO_PD_ value (0.5 mg/L) was 8-fold greater than ciprofloxacin MIC breakpoint against *Haemophilus influenzae* (0.06 mg/L), but was equivalent to PK/PD breakpoints (0.25–0.5 mg/L) of other fluoroquinolones such as ofloxacin moxifloxacin (42). Of note, danofloxacin ECOFF for *H. parasuis* (4 mg/L) was higher compared to the corresponding CO_PD_ (0.5 mg/L) in this study. The over-estimated ECOFF value could be due to other unknown resistance mechanisms and the limited number of strains collected. A similar cutoff value (≥2 mg/L) was observed for enrofloxacin against *Haemophilus somnus* (13). This result suggested that danofloxacin at 2.5 mg/kg may be insufficient to combat swine respiratory infections due to *H. parasuis* with high-level MICs of > 4 mg/L. Indeed, a higher danofloxacin dosage of 13.36 mg/kg is required to achieve a PTA of > 90% for bactericidal effect against the overall *H. parasuis* isolates collected in this study.

## Conclusion

In summary, we have demonstrated a large potentiation effect of serum on the potency of danofloxacin for *H. parasuis*. Compared with *P. multocida*, the PAEs and PA-SMEs of danofloxacin were substantially longer for *H. parasuis*. The PK/PD targets and cutoff values identified in this study will be useful in guiding the optimum dosing regimen design for danofloxacin in the context of specific PK exposure and MIC distribution, and in the development of clinical breakpoints for the treatment of SRD involving these pathogens.

## Data Availability Statement

The original contributions presented in the study are included in the article/Supplementary Material, further inquiries can be directed to the corresponding author/s.

## Ethics Statement

The animal study was reviewed and approved by the South China Agricultural University (SCAU) Institutional Ethics Committee (Approval No. 2018014). Written informed consent was obtained from the owners for the participation of their animals in this study.

## Author Contributions

Y-FZ and X-PL designed this study. Y-FZ and ZS wrote the manuscript. ZS, R-LW, J-GL, C-YN, X-AL, and Y-YF carried out the experiments. JS and Y-HL analyzed the data. All authors read and approved the final manuscript.

## Funding

This work was supported by the Laboratory of Lingnan Modern Agriculture Project (NT2021006), the Foundation for Innovative Research Groups of the National Natural Science Foundation of China (32121004), National Natural Science Foundation of China (31902318), the Local Innovative and Research Teams Project of Guangdong Pearl River Talents Program (2019BT02N054), Program for Changjiang Scholars and Innovative Research Team in University of Ministry of Education of China (IRT_17R39), the Found for Fostering Talents of College of Veterinary Medicine of South China Agricultural University (5500A17003), and the Innovative Team Project of Guangdong University (2019KCXTD001).

## Conflict of Interest

The authors declare that the research was conducted in the absence of any commercial or financial relationships that could be construed as a potential conflict of interest.

## Publisher's Note

All claims expressed in this article are solely those of the authors and do not necessarily represent those of their affiliated organizations, or those of the publisher, the editors and the reviewers. Any product that may be evaluated in this article, or claim that may be made by its manufacturer, is not guaranteed or endorsed by the publisher.
