# Supplementary material for: Comparison of PK/PD Targets and Cutoff Values for Danofloxacin Against Pasteurella multocida and Haemophilus parasuis in Piglets
Source: Front Vet Sci. 2022 Feb 2;9:811967. doi: 10.3389/fvets.2022.811967 (PMC8847440; doi:10.3389/fvets.2022.811967)
Supplement: Supplementary file 1 [file Data_Sheet_1.PDF]

Supplementary Materials for

Comparison of PK/PD targets and cutoff values for danofloxacin against *Pasteurella multocida* and *Haemophilus parasuis* in piglets

The Supplementary File Includes:

Methods. Sample processing and LC-MS/MS method for danofloxacin

Table S1. Summary of minimal inhibitory concentrations (MICs) and frequency distributions of danofloxacin against *P. multocida* (n = 931) and *H. parasuis* (n = 263) isolated from pigs in United States, Canada, Spain and China.

Figure S1. Potentiation effects of serum on danofloxacin activity.

Figure S2. Danofloxacin PAEs and PA-SMEs for additional *P. multocida* (A) and *H. parasuis* (B, C) strains.

Figure S3. Danofloxacin *ex vivo* activity and PK/PD relationships for additional *P. multocida* and *H. parasuis* strains.

Supplementary Materials & Methods

Sample processing and LC-MS/MS method for danofloxacin

A 50 μL aliquot of porcine serum was transferred into a capped centrifuge tube, and then mixed with 50 μL of acetonitrile. After vortexing (1 min) and centrifuging (12000×g, 10 min), the supernatant was filtered through a 0.22 μm nylon syringe filter and collected into a vial for concentration determination. Serum drug concentrations were determined by a HPLC-ESI-MS/MS system (Agilent 1200 HPLC system; Agilent Technologies, Santa Clara, CA, USA; API 4000 triple quadrupole mass spectrometer; Applied Biosystems, Carlsbad, CA, USA) equipped with a short column (Waters Symmetry C18, 2.1×100 mm, 3.5 μm) as previously described [1, 2]. The injection volume was 5 μL, and column temperature was maintained at 30 °C. The mobile phase consisted of (A) acetonitrile and (B) 0.1% formic acid in water using a gradient elution with a flow rate of 250 μL/min: 0-0.5 min (5% A), 0.5-1.5 min (5-85% A), 1.5-3.5 min (85% A), 3.5-4.0 min (85-5% A), 4.0-11 min (5% A). The mass conditions were as follows: ionspray voltage, 5500 V; curtain gas, 10 psi; nebulizer gas, 25 psi; source temperature, 500 °C. Multiple reaction monitoring (MRM) transitions from m/z 358.3→340.2 and 358.3→314.2 were chosen for danofloxacin in the positive mode. The danofloxacin was verified and quantified using a calibration curve derived from an analytical standard purchased from Sigma-Aldrich. The coefficient of determination (R^2) was above 0.980 in the linear concentration range of 0.005 to 0.5 μg/mL. All samples that had concentrations above 0.5 μg/mL were diluted proportionally prior to extraction with acetonitrile. The limits of quantification (LOQ) and detection (LOD) were 0.002 and 0.001 μg/mL, respectively. The mean extraction recoveries from five replicate assays ranged from 93.8% to 102.6% at the spiked drug concentrations of 0.01 to 0.2 μg/mL. Intraday and interday variabilities in precision (expressed as the coefficient of variation) were 3.72-7.99% and 5.33-9.73%, respectively.

Table S1. Summary of minimal inhibitory concentrations (MICs) and frequency distributions of danofloxacin against *P. multocida* (n=931) and *H. parasuis* (n=263) isolated from pigs in United States, Canada, Spain and China. [a]

Sources	Year	Area	No.	MIC_{50}	MIC_{90}	Count of isolates and danofloxacin MIC frequency distribution (% of isolates)															
						**0.004**	**0.008**	**0.016**	**0.03**	**0.06**	**0.125**	**0.25**	**0.5**	**1**	**2**	**4**	**8**	**16**	**32**	**64**	**128**
P. multocida **(n = 931)**																					
Sweeney[3]	2011-2015	USA Canada	855	0.016	0.03			469	331	51	3	1									
Dolores CID[4]	2001-2009	Spain	69	0.125	0.5						49	6	9	1	4						
This study	2015-2018	China	7	NA	NA			1		1	1	1	1	1		1					
Total	2001-2018	NA	931	0.016	0.06			470	331	52	53	8	10	2	4	1					
Percent %	2001-2018	NA	%	NA	NA			50.5	35.5	5.6	5.7	0.9	1.1	0.2	0.4	0.1					
H. parasuis **(n = 263)**																					
This study	2015-2020	China	263	0.25	4	5	5	11	13	24	34	45	36	34	25	12	5	2	2	4	6
Percent %	2015-2020	NA	%	NA	NA	1.9	1.9	4.2	4.9	9.1	12.9	17.1	13.7	12.9	9.5	4.6	1.9	0.8	0.8	1.5	2.3

[a] No. = the number of isolates tested; MIC_{50} and MIC_{90} = danofloxacin concentration that inhibits 50% or 90% of the bacterial population; NA = not applicable.

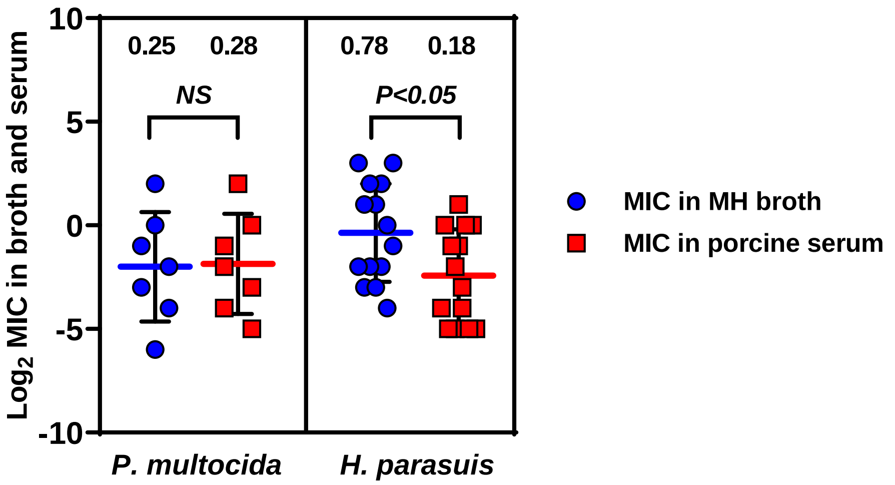

Figure S1. Potentiation effects of serum on danofloxacin activity. MICs of danofloxacin determined in MH or HTM broth and porcine serum against *P. multocida* (left panel; n = 7) and *H. parasuis* (right panel; n = 14). Each data point represents the MIC of a single isolate, and the horizontal lines indicate the geometric mean values for each group. *P<0.05*, significantly different from MICs in HTM versus porcine serum for *H. parasuis* (paired Student *t*-test); *NS*, no significance.

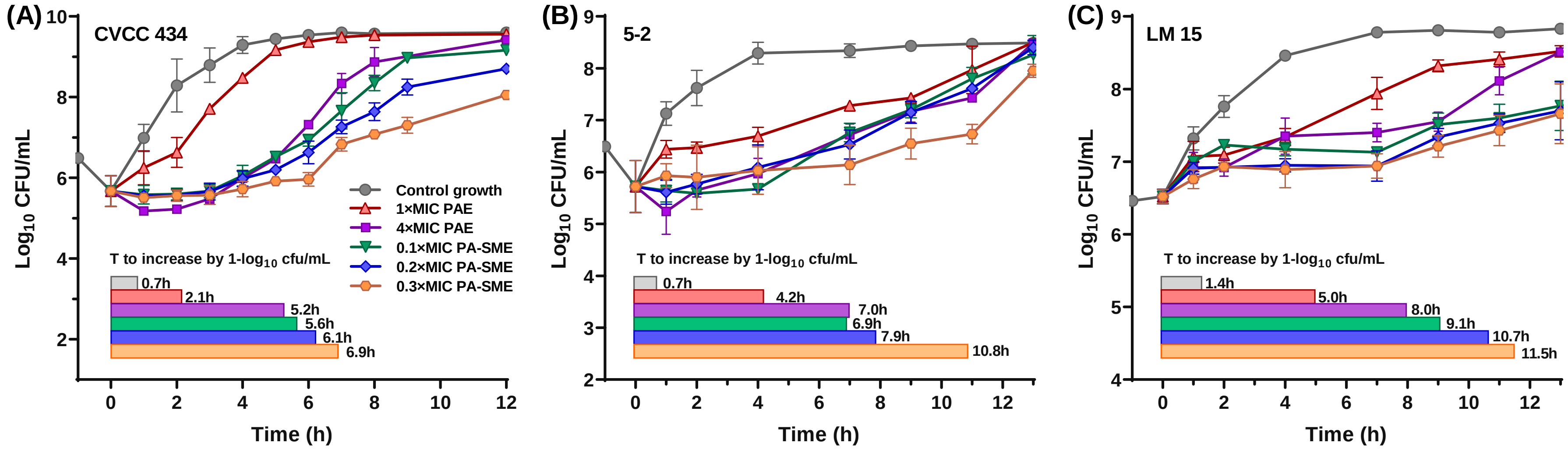

Figure S2. Danofloxacin PAEs and PA-SMEs for additional *P. multocida* (A) and *H. parasuis* (B, C) strains. PAEs were measured after initial exposure to danofloxacin at 1× and 4× MICs against *P. multocida* (panel A; strain CVCC 434) and *H. parasuis* (panel B-C; strain 5-2 and LM15). PA-SMEs were measured after initial exposure to danofloxacin at 4×MICs. The black horizontal bars represent the time that required bacterial counts to increase by 1.0-log_{10}cfu/mL after drug removal (PAE) or at the sub-MIC phase (PA-SME).

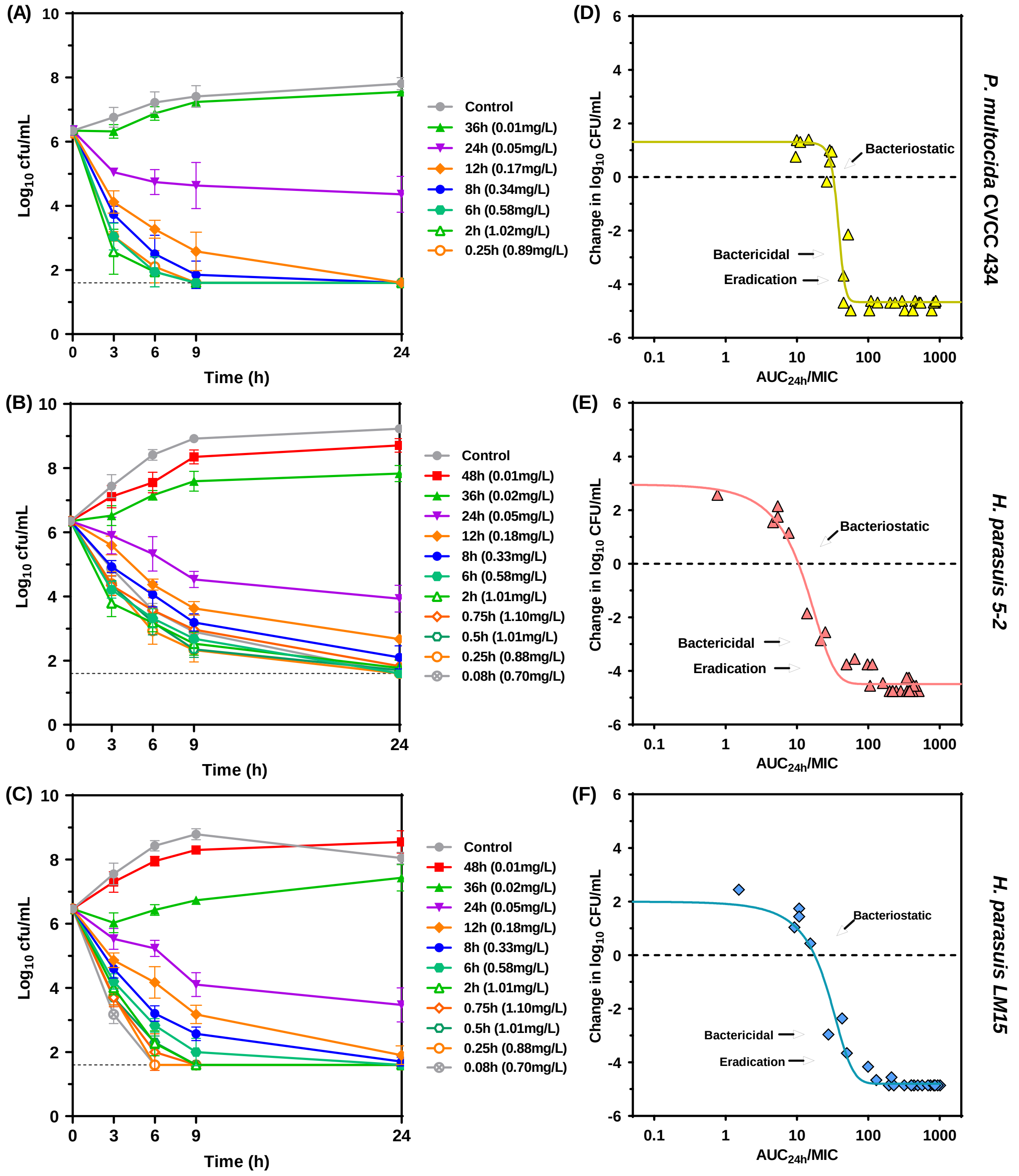

Figure S3. Danofloxacin *ex vivo* activity and PK/PD relationships for additional *P. multocida* and *H. parasuis* strains. (**A-C**) *Ex vivo* time-kill curves of danofloxacin against *P. multocida* (A; strain CVCC 434; MIC_{serum}=0.03 mg/L) and *H. parasuis* (B-C; strain 5-2, MIC_{serum}=0.06 mg/L; strain LM15, MIC_{serum}=0.03 mg/L) in serums of piglets receiving intramuscular injection of danofloxacin at 2.5 mg/kg. Numerical values on right brackets are the mean concentrations of danofloxacin in serums collected at different time points post-dosing. (**D-F**) Correlation plots between *ex vivo* activity and AUC_{24h}/MIC ratio of danofloxacin against the additional *P. multocida* and *H. parasuis* strains. The fitting curves represent the predicted values based on the sigmoid E_{max} model, and the points represent values of individual serum samples collected from 0 to 48 h.

References for Supplementary Materials

1. Lopez BS, Giguere S, Berghaus LJ, Mullins MA, Davis JL: Pharmacokinetics of danofloxacin and N-desmethyldanofloxacin in adult horses and their concentration in synovial fluid. Journal of Veterinary Pharmacology and Therapeutics 2015, 38(2):123-129.
2. Annunziata L, Visciano P, Stramenga A, Colagrande MN, Campana G, Scortichini G, Migliorati G, Compagnone D: Development and validation of a method for the determination of quinolones in muscle and eggs by liquid chromatography-tandem mass spectrometry. Food Analytical Methods 2016, 9(8):2308-2320.
3. Sweeney MT, Lindeman C, Johansen L, Mullins L, Murray R, Senn MK, Bade D, Machin C, Kotarski SF, Tiwari R *et al*: Antimicrobial susceptibility of *Actinobacillus pleuropneumoniae*, *Pasteurella multocida*, *Streptococcus suis*, and *Bordetella bronchiseptica* isolated from pigs in the United States and Canada, 2011 to 2015. Journal of Swine Health and Production 2017, 25(3):106-120.
4. Cid D, Fernández-Garayzábal JF, Pinto C, Domínguez L, Vela AI: Antimicrobial susceptibility of *Pasteurella multocida* isolated from sheep and pigs in Spain. Acta Veterinaria Hungarica 2019, 67(4):489-498.
